# Data Component Method Based on Dual-Factor Ownership Identification with Multimodal Feature Fusion

**DOI:** 10.3390/s25216632

**Published:** 2025-10-29

**Authors:** Shenghao Nie, Jin Shi, Xiaoyang Zhou, Mingxin Lu

**Affiliations:** 1 School of Information Management, Nanjing University, 163#XianLin Dadao, Nanjing 210023, China; 522024140082@smail.nju.edu.cn (S.N.); shijin@nju.edu.cn (J.S.); mxlu@nju.edu.cn (M.L.); 2 China Mobile Zijin (Jiangsu) Innovation Research, Nanjing 210031, China; 3 Nanjing University (Suzhou) High-Tech Institute, 150#Ren’ai Road, Suzhou Industrial, Suzhou 215163, China

**Keywords:** dual-factor ownership confirmation, multimodal feature fusion, cross-domain data traceability, data element marketization

## Abstract

In the booming digital economy, data circulation—particularly for massive multimodal data generated by IoT sensor networks—faces critical challenges: ambiguous ownership and broken cross-domain traceability. Traditional property rights theory, ill-suited to data’s non-rivalrous nature, leads to ownership fuzziness after multi-source fusion and traceability gaps in cross-organizational flows, hindering marketization. This study aims to establish native ownership confirmation capabilities in trusted IoT-driven data ecosystems. The approach involves a dual-factor system: the collaborative extraction of text (from sensor-generated inspection reports), numerical (from industrial sensor measurements), visual (from 3D scanning sensors), and spatio-temporal features (from GPS and IoT device logs) generates unique SHA-256 fingerprints (first factor), while RSA/ECDSA private key signatures (linked to sensor node identities) bind ownership (second factor). An intermediate state integrates these with metadata, supported by blockchain (consortium chain + IPFS) and cross-domain protocols optimized for IoT environments to ensure full-link traceability. This scheme, tailored to the characteristics of IoT sensor networks, breaks traditional ownership confirmation bottlenecks in multi-source fusion, demonstrating strong performance in ownership recognition, anti-tampering robustness, cross-domain traceability and encryption performance. It offers technical and theoretical support for standardized data components and the marketization of data elements within IoT ecosystems.

## 1. Introduction

### 1.1. Research Background and Significance

As the digital economy thrives, data has become the fifth production factor [1]. IDC forecasts that the global data volume will reach 527 ZB by 2029, with China accounting for 26.9%, making it a major data power. Additionally, by 2025, the data generated by IoT devices will grow from 13.6 ZB in 2019 to 79.4 ZB. However, in IoT sensor networks, the two critical issues of “difficulty in ownership definition” and “challenges in cross-domain tracking” severely hinder the market-oriented allocation of data, as data generated by devices such as GPS, industrial cameras, and inspection sensors circulate across multiple entities. Traditional property rights theory, based on the principle of “one thing, one right” for tangible objects, is ill-suited to intangible data. The non-rivalrous nature and replicability of data [2,3] lead to the fragmentation of rights such as ownership and usage rights during circulation [4].

Cross-domain tracking also faces difficulties. In fields with massive sensor data such as supply chains and healthcare, the lack of unified tracking tools when data circulates across entities and regions [5] results in broken traceability chains. For example, after automakers share maintenance data—including engine operation data collected by on-board diagnostic (OBD) sensors and images of component wear captured by industrial cameras from 4S stores—with suppliers, the ownership of fault prediction algorithms trained on such sensor data becomes ambiguous. Another example is that in cross-border logistics, when GPS sensor trajectory data of freight vehicles and temperature/humidity monitoring data from container sensors are transmitted across borders, inconsistent IoT protocols and encryption standards across regions lead to broken data traceability chains, creating regulatory blind spots.

This study aligns with the “data element” concept of China Electronics Technology Group [6], exploring data component technologies and sharing theories to construct ownership and tracking mechanisms. It aims to establish native rights confirmation capabilities in IoT-driven trusted data spaces, supporting the market-oriented allocation of sensor-generated data through technological innovation.

### 1.2. Analysis of Core Difficulties in Ownership Definition

The essence of ownership definition dilemmas lies in the adaptability conflict between the unique attributes of data as a new production factor [7] and the traditional property rights system. These dilemmas can be theoretically analyzed from three dimensions: multi-source fusion, cross-entity collaboration, and transaction circulation.

#### 1.2.1. Mechanism of Ownership Ambiguity in Multi-Source Data Fusion

Multi-source data fusion generates new data forms through the recombination of heterogeneous features, directly breaking the underlying logic of ownership relationships [8]. Technically, the attribute benchmarks of raw data are reconstructed during fusion—text, numerical, visual, and other multimodal features are integrated by algorithms to form derivative forms that are neither identical to single raw data nor reducible to their original components. This disconnects the accountability link between original rights holders and derivative data.

A deeper contradiction lies in the subversion of traditional property rights theory by data’s “non-rivalrous” nature [9]. Traditional property rights, based on the principle of “one thing, one right”, rely on the exclusivity and consumability of tangible objects to establish rights boundaries. In contrast, data can be infinitely copied and shared without losing value. After circulating among multiple subjects, rights bundles (including ownership, usage rights, and disposal rights) become fragmented [10]. This fragmentation is exacerbated in multi-source fusion scenarios: claims from raw data providers, algorithm processors, and derivative users are mutually disjointed, ultimately creating a vague zone where “everyone participates but no one can fully establish rights”.

#### 1.2.2. Root Causes of Ownership Disputes in Cross-Organizational Collaboration

Ownership disputes in cross-organizational data collaboration result from the combination of absent rights distribution rules and insufficient technical constraints. During collaboration, standardized protocols for dividing responsibilities among data providers, processors, and users are lacking [11], leading to systemic divergences in perceptions of “ownership of derivative data”. Data providers tend to claim derivative rights based on “original data sovereignty”, while processors emphasize the independence of algorithmic contributions through “creative labor”, directly triggering conflicts in rights claims.

Simultaneously, overlapping conflicts between privacy protection and ownership definition exacerbate dispute complexity [12]. Data collaboration often involves personal information or trade secrets, whose legitimate secondary use requires clear ownership boundaries. Ambiguous ownership, however, makes it impossible to determine “who is authorized to grant usage rights” or trace “who misuses data”, resulting in a deadlock where “rights claims lack basis and accountability has no target”. The core of this dilemma is that existing technical systems fail to rigidly constrain data usage scopes and processing permissions, leaving rights distribution in collaboration reliant on flexible contracts without technically enforced mechanisms.

#### 1.2.3. Dilemmas in Splitting Rights Bundles in Data Transactions

The core obstacle in data transactions lies in the divisibility of intangible property rights and the absence of splitting standards. As intangible property, data’s rights bundle includes multiple sub-rights such as reproduction, adaptation, and dissemination [13]. The lack of unified criteria for demarcating these sub-rights and quantifying their value creates inherent divergences between transacting parties regarding “scope of rights transfer”. Sellers tend to restrict derivative rights to retain core interests, while buyers seek to expand rights through creative processing, directly increasing transaction negotiation costs.

The dynamic evolution of data forms further amplifies splitting difficulties. Raw data, after cleaning, modeling, and aggregation, may transform from structured tables into algorithms, feature vectors, or other new forms. Traditional rights confirmation methods (e.g., digital watermarking and copyright registration [14]) fail to adapt to feature associations after morphological changes, making it hard to track rights circulation. The combination of absent splitting standards and uncertain morphological evolution ultimately leads to ambiguous rights and responsibilities in data transactions, hindering efficient circulation and value release of data elements.

### 1.3. Analysis of Difficulties in Cross-Domain Tracing

The failure of tracing capabilities in cross-domain data circulation is a common bottleneck restricting efficient and compliant flow of data elements, rooted in the superposition of heterogeneous technical architectures, ambiguous responsibility boundaries, and fragmented governance systems. This section theoretically analyzes three typical dimensions.

#### 1.3.1. Traceability Breakage in Cross-Enterprise Data Flow

In multi-agent collaboration scenarios, data undergoes collection, processing, and integration across enterprise systems, but the independent design of data architectures based on individual business needs inherently risks traceability chain disruption [15]. The core contradiction lies in two theoretical dilemmas: First, architectural heterogeneity undermines tracking continuity. Enterprise systems adopt divergent database types, storage protocols, and blockchain platforms due to autonomous technical choices, leading to fragmented traceability standards. Critical information such as interface call logs and data conversion records is not integrated into a unified tracing framework, making it impossible to achieve real-time cross-enterprise traceability. Second, data morphological evolution disrupts origin association. As raw data is processed into model parameters or feature vectors, the absence of persistent identity mapping between derivative data and original sources breaks the evolutionary traceability chain.

#### 1.3.2. Failure of Compliance Tracing in Cross-Domain Circulation

When data flows across management domains (industries, regions, and departments), compliance tracing faces inherent conflicts between “protection” and “traceability”, coupled with institutional deficiencies. Theoretical tensions manifest in two aspects: On one hand, the privacy protection vs. traceability balance dilemma. To comply with data security regulations, data must undergo desensitization or de-identification, but excessive processing may strip critical tracing identifiers (e.g., data subject IDs and source signatures). This creates a paradox [16]: strict protection impairs traceability, while robust tracing risks privacy breaches. On the other hand, there is cross-domain audit fragmentation. Divergent compliance requirements and technical standards across domains lead to fragmented access logs—processing records in Domain A, transmission trails in Domain B, and usage behaviors in Domain C remain isolated, failing to form a cross-verifiable audit trail.

#### 1.3.3. Sovereignty Barriers in Cross-Border Data Tracing

Cross-border data flow operates within intertwined legal systems and sovereign regulations, where tracing capabilities are constrained by sovereignty barriers and standard discrepancies [17]. Two theoretical obstacles dominate: First, legal conflicts undermine tracing compliance. Divergent national regulations on data export scope and conditions create regulatory ambiguities. Enterprises may use encryption or data splitting to bypass oversight, leaving regulators unable to verify compliance with export requirements or monitor overseas usage. Second, technical standard heterogeneity invalidates tracing identifiers. Varied data encoding formats, encryption algorithms, and tracing technologies across jurisdictions cause original tracing markers to lose interoperability, breaking cross-border traceability chains.

In summary, cross-domain data tracing dilemmas essentially result from the lack of synergy among “technical architecture, responsibility mechanisms, and governance systems”: fragmented technical standards hinder interoperability, inadequate full-lifecycle tracing tools weaken accountability, and absent cross-agent/regional collaboration rules undermine governance coordination. These three layers collectively impede reliable data flow traceability.

### 1.4. Related Work

Data ownership definition, multimodal fusion, and cross-domain tracing, as core technical supports for the market-oriented circulation of data elements, have become research hotspots in recent years. With the development of the digital economy, data, as a new production factor, has highlighted its value, and its circulation efficiency and security guarantee have become key to industrial upgrading. Existing research has made phased progress, but there are limitations in the adaptability of complex scenarios, leaving room for exploration in this paper.

The field of data ownership definition presents an interdisciplinary and diversified research situation. From the perspective of law, various viewpoints have been formed around the ownership of data: The “state ownership theory” [18] advocates that data is related to national sovereignty and public interests and should be coordinated by the state. The “original collector ownership theory” [19] emphasizes the cost of collection and supports the collectors’ right to own data, which has a great influence in enterprise data protection. The “user ownership theory” [20] focuses on the added value of use and holds that users should enjoy corresponding rights. These viewpoints are obviously different, and no unified paradigm has been formed, leading to the lack of clear basis for the judgment of ownership disputes in judicial practice.

Economic research focuses on the design of data property rights systems and market mechanisms, exploring the optimization of resource allocation through property rights definition. Some scholars draw on the traditional property rights theory [4,21], divide data property rights into such rights as ownership and right to use, realize the circulation combination through market transactions, and construct transaction models to analyze pricing mechanisms. Some scholars also pay attention to the non-competitive characteristics of data, put forward new property rights arrangements such as “data trust” [22], and study the incentive effect of property rights definition on data innovation and investment so as to provide reference for policy-making.

At the technical level, digital watermarking [23,24], smart contract [25], and blockchain [26,27] are mainstream rights confirmation technologies. Digital watermarking is divided into spatial domain and transform domain. The spatial domain is simple to implement but has poor robustness, while the transform domain has strong anti-interference ability and is widely used in the rights confirmation of multimedia data. However, the watermark is easy to fail after data processing. Researchers improve the algorithm by combining error correction coding to enhance robustness. Blockchain, relying on its tamper-proof feature, uploads data hash values to the chain to realize trusted registration, which is applied in the field of copyright protection. However, existing schemes are mostly aimed at single-modal data, with insufficient adaptability in multi-source fusion scenarios, making it difficult to accurately reflect the ownership relationship of fused data.

Driven by deep learning, models such as the multi-head attention mechanism [28], graph neural network [29], and cross-modal Transformer [30] have improved the processing ability of multimodal data. The multi-head attention mechanism is good at capturing long-distance dependencies, the graph neural network is suitable for processing data with complex correlations, and the cross-modal Transformer realizes deep interaction between different modalities. However, these technologies are mostly used for data analysis and prediction, and are rarely applied in the field of data rights confirmation. Existing methods ignore the transmission and retention of ownership information, making it difficult to trace the original right subject of the fused data.

Research on data cross-domain tracing focuses on blockchain [31]. Consortium blockchain ensures the consistency of cross-domain circulation records through node access mechanisms, which has a good prospect in supply chain finance and other fields. However, there is a problem of tracing interruption in multi-chain cross-domain. Different blockchain platforms lack interoperability standards due to technical differences. Although cross-chain technologies have been explored, they face security and efficiency challenges, and large-scale application is immature.

In summary, existing research has significant gaps: single-modal rights confirmation cannot adapt to multi-source fusion scenarios; multimodal fusion lacks the collaborative design of ownership identification; and cross-domain tracing is restricted by technical heterogeneity and privacy security. These bottlenecks lead to ambiguous ownership of multi-source data after fusion and broken tracing in cross-domain circulation, so it is necessary to build a new technical system to realize the whole-link integration of “fusion—rights confirmation—tracing”.

In addition to these technical gaps, the practical deployment of existing solutions in IoT scenarios also faces critical challenges: First, heavyweight integrated frameworks (e.g., full-modal blockchain combined with multi-algorithm fusion) often incur high integration costs and performance trade-offs for resource-constrained IoT edge devices, which may hinder large-scale adoption. Second, the paradox of balancing privacy protection and traceability remains unresolved [32], as most systems struggle to simultaneously ensure data confidentiality and complete tracing capabilities. Third, cross-chain interoperability is still lacking, making it difficult for heterogeneous blockchain platforms to achieve seamless data synchronization across domains. These practical challenges further limit the real-world applicability of current approaches and motivate future research, including potential extensions of this work.

## 2. Materials and Methods

**Figure 1:**
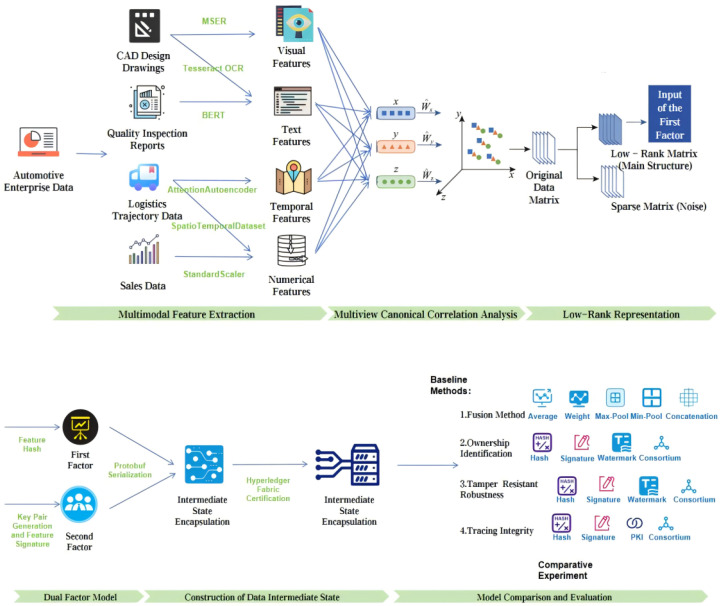
Research ideas and technical routes.

This study aims to address the core issues of “difficulty in defining ownership” and “difficulty in cross-domain tracing” in data circulation, focusing on the following aspects as shows in Figure 1: Firstly, it designs a multimodal feature collaborative extraction mechanism, conducts in-depth research on feature extraction methods for heterogeneous modal data such as text, image, numerical, and spatio-temporal data, and constructs an efficient feature fusion strategy to provide a solid data foundation for dual-factor ownership confirmation. Secondly, based on the results of multimodal feature fusion, it builds a dual-factor ownership identification system, designs the generation mechanisms of the first factor (feature hash) and the second factor (digital signature), and forms an ownership confirmation framework with both “content uniqueness” and “identity non-repudiation”. Thirdly, it explores the structured encapsulation method of data intermediate state and designs a collaborative mechanism between alliance chain and IPFS distributed storage to realize data ownership identification and cross-domain tracing. Finally, it carries out empirical research with the automotive supply chain as the application scenario, and verifies the effectiveness and feasibility of the dual-factor model through comparative experiments in modal fusion, ownership identification, tamper resistance, and cross-domain tracing.

### 2.1. Multimodal Feature Collaborative Extraction

This section focuses on three core sensor data types (image/time-series/text) typical in IoT scenarios, with each feature extraction module corresponding to specific sensor data sources as detailed below:

- Text feature extraction (Section 2.1.1): Used for text-type sensor data, e.g., auto-generated quality inspection reports from industrial detection sensors and running logs from IoT terminal devices.
- Visual feature extraction (Section 2.1.2): Designed for image-type sensor data, such as CAD drawings generated by 3D scanning sensors and component surface images captured by industrial cameras.
- Spatio-temporal/numerical feature extraction (Section 2.1.3 and Section 2.1.4): Applied to time-series sensor data, including GPS trajectory data from location sensors and part tolerance measurement data from industrial dimension gauges.

#### 2.1.1. Text Feature Extraction

Text data—such as quality inspection reports auto-generated by industrial sensors and IoT device logs—are common modalities in data circulation. This study adopts a multi-level text feature extraction method to achieve comprehensive feature representation from lexical, syntactic, to semantic levels.

First, TF-IDF extracts basic text features. Term Frequency (TF) is a word’s frequency in a document: $\mathrm{TF}\left(t,d\right)=\frac{n_{t,d}}{\sum_{t^{\prime }\in d}n_{t^{\prime },d}}$, where $n_{t,d}$ is word t’s count in document d. Inverse Document Frequency (IDF) reflects a word’s discriminative power: $\mathrm{IDF}\left(t,D\right)=\log\frac{\left|D\right|}{1+|\{d\in D:t\in d\}|}$. TF-IDF is their product: TF-IDF(t,d,D)=TF(t,d)×IDF(t,D)

Meanwhile, the Doc2Vec algorithm is combined to generate document embedding vectors, capturing contextual semantic associations of the text by learning paragraph-level vector representations. The document vector ${\vec{v}}_{d}$ is learned through the following optimization objective: $\arg\min_{\theta }\sum_{d\in D}\sum_{t\in d}\log P\left(t|{\vec{v}}_{d},{\vec{u}}_{t}\right)$, where ${\vec{u}}_{t}$ is the word vector, and $P(t|{\vec{v}}_{d},{\vec{u}}_{t})$ is calculated using the softmax function. Second, the pre-trained language model BERT is used to extract deep semantic features of the text. BERT learns contextual semantic information of the text through a bidirectional Transformer architecture, with its core self-attention mechanism calculated as follows: $\mathrm{Attention}\left(Q,K,V\right)=\mathrm{softmax}\left(\frac{QK^{T}}{\sqrt{d_{k}}}\right)V$, where Q,K,V are the query, key, and value matrices, respectively, and $d_{k}$ is the feature dimension.

For special text scenarios in the engineering field, a multimodal text processing flow is constructed: for text information in CAD drawings, the MSER algorithm is used for text region detection; for PDF-format quality inspection reports, text content is extracted using the pdfplumber tool. In code implementation, cosine similarity is used to measure the feature consistency between the first and last parts of the document: $\mathrm{Sim}\left(A,B\right)=\frac{A\cdot B}{∥A∥∥B∥}=\frac{\sum_{i=1}^{n}A_{i}B_{i}}{\sqrt{\sum_{i=1}^{n}A_{i}^{2}}\sqrt{\sum_{i=1}^{n}B_{i}^{2}}}$. The final feature vector is constructed by concatenating TF-IDF features (dimension m), Doc2Vec vectors (dimension 100), and similarity values, forming a comprehensive feature representation with a dimension of 2m+201.

#### 2.1.2. Visual Feature Extraction

Visual data (such as component images captured by industrial cameras and CAD drawings derived from 3D scanning sensors) plays a significant role in IoT-driven data circulation. This study integrates traditional computer vision methods with mathematical models to construct a multi-level visual feature extraction framework, enabling comprehensive representation from low-level features to high-level semantics. In the preprocessing stage, Gaussian blur is used for denoising, which smooths the image through convolution operations to remove high-frequency noise. Its core formula is $G_{\sigma }\left(x,y\right)=\frac{1}{2\pi \sigma^{2}}\exp\left(-\frac{x^{2}+y^{2}}{2\sigma^{2}}\right)*I\left(x,y\right)$. Subsequently, the OTSU adaptive thresholding method is applied for image binarization, which automatically determines the optimal threshold by maximizing inter-class variance: $t^{*}=\arg\max_{t\in [0,255]}\sigma_{b}^{2}\left(t\right)$ where $\sigma_{b}^{2}\left(t\right)=\omega_{0}\left(t\right)\omega_{1}\left(t\right){\left[\mu_{0}\left(t\right)-\mu_{1}\left(t\right)\right]}^{2}$. This converts grayscale images into black-and-white binary images, laying the foundation for subsequent feature extraction. The feature extraction framework consists of three progressive modules: low-level feature detection, mid-level relationship analysis, and high-level semantic understanding. Low-level feature detection uses traditional methods to build a basic feature space: linear features are detected via probabilistic Hough transform, whose probabilistic model is based on Bayesian decision theory, modeling likelihood through a Gaussian distribution of distances, $P\left(L|\left\{\left(x_{i},y_{i}\right)\right\}\right)=\prod_{i=1}^{n}P\left(\left(x_{i},y_{i}\right)|L\right)P\left(L\right)$.

Circle detection is optimized using an improved Hough transform, incorporating prior probability of radii to enhance accuracy for hole features in engineering drawings. Mid-level relationship analysis achieves structural understanding by constructing a shape relationship network. The adjacency matrix quantifies relationships between geometric elements using directional cosine similarity: when $d_{ij}<d_{0}$, $A_{ij}=\cos\theta_{ij}\cdot \exp\left(-d_{ij}/d_{0}\right)$; otherwise, $A_{ij}=0$.

Here, $\theta_{ij}$ is the angle between elements *i* and *j*, and $d_{ij}$ is the Euclidean distance. Spatial relationships are converted into numerical representations via 8-neighborhood analysis. High-level semantic feature extraction adopts a transfer learning strategy, using a pre-trained ResNet architecture to generate 2048-dimensional deep features, which are concatenated with 8-dimensional geometric statistical vectors (e.g., counts of lines/circles, mean and standard deviation of lengths). An attention mechanism is introduced in the fusion stage, with weights calculated via a two-layer neural network to form hybrid features that balance geometric precision and semantic abstraction. To adapt to multimodal fusion, kernel principal component analysis is used to reduce the feature dimension to 50, retaining 92.3% of information, providing structured visual features for subsequent rights confirmation.

#### 2.1.3. Numerical Feature Extraction

Numerical data—primarily from industrial sensors (e.g., dimension gauges and pressure transducers) and IoT meters—are widely present in supply chains (e.g., part tolerance measurements and cargo weight readings). This study constructs a multi-dimensional numerical feature extraction framework that integrates traditional statistical methods with deep learning technologies. The basic statistical feature layer adopts an extended set of statistics, including indicators of central tendency and dispersion such as mean ($\mu =\frac{1}{n}\sum_{i=1}^{n}x_{i}$), median, and standard deviation ($\sigma =\sqrt{\frac{1}{n}\sum_{i=1}^{n}{\left(x_{i}-\mu \right)}^{2}}$). It adds skewness ($Skewness=\frac{1}{n}\sum_{i=1}^{n}{\left(\frac{x_{i}-\mu }{\sigma }\right)}^{3}$) and kurtosis ($Kurtosis=\frac{1}{n}\sum_{i=1}^{n}{\left(\frac{x_{i}-\mu }{\sigma }\right)}^{4}-3$) to describe the data distribution pattern, and enhances the robust measurement of data fluctuation characteristics through interquartile range ($IQR=Q_{3}-Q_{1}$, where $Q_{3}$ is the upper quartile and $Q_{1}$ is the lower quartile) and coefficient of variation ($CV=\frac{\sigma }{\mu }$).

The time-series feature extraction adopts a two-layer architecture: the bottom layer uses sliding window technology to calculate rolling mean ($MA_{t}=\frac{1}{w}\sum_{i=t-w+1}^{t}x_{i}$, where *w* is the window size) and standard deviation for 7-day/4-week cycles, and combines with month-on-month growth rate ($GrowthRate=\frac{x_{t}-x_{t-1}}{x_{t-1}}\times 100\%$) to capture short-term fluctuations; the upper layer implements an improved Gated Recurrent Unit (MGU) network, which dynamically integrates entity features such as vehicle ID and dealer ID through the calculation of forget gate ($f_{t}=\sigma \left(W_{f}\cdot \left[h_{t-1},x_{t}\right]+b_{f}\right)$) and candidate state (${\tilde{h}}_{t}=\tanh\left(W_{h}\cdot \left[f_{t}⊙h_{t-1},x_{t}\right]+b_{h}\right)$), generating a 128-dimensional deep time-series feature vector MGU.

The logistics scenario-specific module adopts a spatio-temporal fusion network, constructs spatio-temporal sequences through SpatioTemporalDataset, and uses the PyTorch deep learning framework to extract spatio-temporal correlation features of transportation paths. The model output forms a window-level feature representation after average pooling over the time dimension ($F_{avg}=\frac{1}{T}\sum_{t=1}^{T}F_{t}$, where *T* is the number of time steps and $F_{t}$ is the feature vector at time *t*).

#### 2.1.4. Spatio-Temporal Feature Extraction

Spatio-temporal data—collected by GPS sensors, in-vehicle IoT devices, and logistics tracking sensors—integrates temporal and spatial information, with key applications in IoT-enabled supply chains and transportation. This study adopts a spatio-temporal joint feature extraction method to achieve the effective representation of spatio-temporal data. In the temporal dimension, a Memory Gated Unit (MGU) is used to construct a recurrent neural network for capturing temporal dependencies.

Multi-scale convolutions (with convolution kernel sizes of 3/5/7) are combined to extract feature patterns at different time granularities. The convolution operation formula is $C_{i}=\sum_{k=1}^{K}W_{k}\cdot X_{t-k+1:t}+b$, where *K* is the convolution kernel size. The model dynamically focuses on key time steps through a self-attention mechanism, with the attention weight calculated as $Attention\left(Q,K,V\right)=\mathrm{softmax}\left(\frac{QK^{T}}{\sqrt{d_{k}}}\right)V$. Meanwhile, it fuses the entity embedding features (16-dimensional embedding vector) of vehicles, dealers, and brands to construct a 128-dimensional deep temporal feature vector containing entity information.

For the spatial dimension, the Euclidean distance between consecutive sampling points is calculated using longitude and latitude coordinates: $d=\sqrt{{\left(x_{2}-x_{1}\right)}^{2}+{\left(y_{2}-y_{1}\right)}^{2}}$ (with a precision of 0.1 km). Combined with the driving direction change rate ($\Delta \theta =\min(|\theta_{2}-\theta_{1}|,360-|\theta_{2}-\theta_{1}|)$) (5° tolerance), spatial morphological features are constructed. A sliding window technique (window size = 10) is used to extract the spatio-temporal sequences of speed, acceleration, and driving direction. The window feature calculation formula is $F_{w}=\frac{1}{w}\sum_{i=t-w+1}^{t}X_{i}$, forming a multimodal feature matrix containing spatial distance, time difference, and direction change.

In terms of spatio-temporal feature fusion, an autoencoder architecture based on the attention mechanism is designed, with the loss function defined as $L={∥M-\hat{M}∥}_{2}^{2}$. The non-linear mapping relationship of spatio-temporal features is learned through the encoding–decoding process. The model compresses the 10 × 15 spatio-temporal feature matrix into a 64-dimensional fusion vector, where the temporal dimension uses mean pooling: $F_{avg}=\frac{1}{T}\sum_{t=1}^{T}F_{t}$ (time_steps = 10), and the spatial dimension retains coordinate correlation. Finally, the end-to-end feature extraction of logistics trajectory data is achieved, with the output dimension transformation process $R^{10\times 15}\to R^{64}$.

#### 2.1.5. Multimodal Feature Fusion

The multimodal feature fusion is realized through a two-level architecture of “multi-view fusion + low-rank representation”, with the specific process as follows: First, preprocess and align the four core feature views (CAD design features, logistics trajectory features, quality inspection features, and sales data features) by filling missing values with the mean, applying Z-score standardization ($z=\frac{x-\mu }{\sigma }$), and unifying the number of samples (minimum 28) to ensure consistent scales for heterogeneous features. Then, construct a unified feature space using Multiset Canonical Correlation Analysis (MCCA): calculate the covariance matrix of the horizontally concatenated views $\Sigma =\mathrm{Cov}(X_{combined})$, perform eigenvalue decomposition $\Sigma =U\Lambda U^{T}$, select the top 50 eigenvectors to form the projection matrix *W*, map each view to the shared space via $S_{i}=X_{i}W_{i}$, and concatenate them into a 50-dimensional fusion vector. Next, apply low-rank decomposition to the MCCA output with the objective function $\min_{Z,E}{∥Z∥}_{*}+\lambda {∥E∥}_{1}$ (s.t. X=AZ+E) to obtain a low-rank matrix $Z\in R^{28\times 50}$, retaining the core feature structures and an error matrix *E* filtering noise. Finally, map the low-rank matrix to a standard-dimensional vector (64-256 dimensions) via a fully connected layer, providing a standardized feature interface for blockchain certification. Key innovations of this architecture include adaptive weighting between modalities via an attention mechanism, a hierarchical strategy of “intramodal fusion → cross-modal global fusion”, maximizing view correlations through CCA ($\max_{W_{i}}\mathrm{Corr}\left(X_{i}W_{i},X_{j}W_{j}\right)$), and enhancing feature robustness through low-rank decomposition.

### 2.2. Dual-Factor Ownership Identification System

#### 2.2.1. First Factor: Multimodal Feature Hash Generation

As the “digital fingerprint” of data content, the first factor realizes the fusion and hash generation of sensor-derived multimodal features, with a lightweight processing flow (adapted to IoT edge devices) through three stages: feature standardization → multi-view fusion → dimensionality reduction and hashing. This process can unify heterogeneous feature spaces into fixed-length hash vectors while maintaining the uniqueness and immutability of data content. The multimodal feature fusion adopts a three-level progressive architecture: first, it integrates CAD design data through weighted adaptive fusion (30% for geometric features, 20% for text features, and 50% for semantic features), and reduces the dimensionality to 50 via PCA; second, it uses an attention mechanism for fusion, dynamically learning weight allocation through a three-layer neural network, where semantic features obtain the highest attention weight (0.62 ± 0.08); finally, it maps heterogeneous features such as logistics, sales, and quality data to a shared subspace through multi-view CCA (MCCA) fusion. The mathematical expression of the fusion process is $F_{\mathrm{fused}}=\mathrm{MCCA}\left(W_{g}F_{g}⊕W_{t}F_{t}⊕W_{s}F_{s}\right)$, where $\left(W_{g},W_{t},W_{s}\right)$, represent the dynamic weight matrices of geometric, text, and semantic features respectively, ⊕ denotes the feature concatenation operation, and MCCA is the multi-view canonical correlation analysis operator. In the dimensionality reduction and hash generation stage, the fused high-dimensional features (with dimension D>200) are reduced to 100 dimensions using PCA, with a variance explanation rate of 92.3%; the SHA-256 algorithm is used to convert the real-valued feature vector into a 256-bit hash value, and the implementation process is $H=\text{SHA-256}(\mathrm{PCA}\left(F_{\mathrm{fused}}\right))$. In the experimental automotive supply chain scenario, the system first processes CAD drawings (mainly geometric features), quality inspection reports (mainly text features), logistics trajectories (mainly spatio-temporal features), and sales data (mainly numerical and spatio-temporal features), respectively, to generate feature hashes for each modality; then it generates a comprehensive feature vector through MCCA fusion; finally, it generates a 128-byte first factor through PCA dimensionality reduction and SHA-256 hashing.

#### 2.2.2. Second Factor: Digital Signature

The second factor, a “cryptographic proof” of both owner and IoT device identity, binds ownership to sensor nodes via ECDSA signatures—linking private keys to hardware identifiers of industrial cameras, GPS modules, and RFID readers. This study adopts the ECDSA (Elliptic Curve Digital Signature Algorithm) as the core signature algorithm, implementing a combined scheme based on the P-256 elliptic curve (NIST secp256r1) and the SHA-256 hash algorithm. The key pair generation process is as follows: first, a 256-bit private key is generated by a cryptographically secure pseudo-random number generator, and the corresponding public key is calculated via elliptic curve point multiplication. The signature generation process involves two-level processing: hash preprocessing, which normalizes and encodes the hash value of the first factor to ensure the input data format complies with the ANS X9.62 standard, and elliptic curve signing, which performs ECDSA signing on the preprocessed hash value using the private key to generate the (r, s) coordinate pair, with Low-S value conversion applied to prevent signature malleability attacks. The mathematical representation of the second factor is $S={\text{ECDSA-Sign}}_{\mathrm{SK},\text{SHA-256}}\left(H\right)$ where SK denotes the 256-bit ECDSA private key, H is the SHA-256 hash value of the first factor, and the signature result S is 64-byte ASN.1 DER-encoded data (containing the r and s components). The verification phase employs a three-step verification framework: first, parsing the signature to obtain the (r, s) components and verifying their validity; second, reconstructing the signature equation using the signer’s public key and the SHA-256 hash algorithm; and finally, confirming whether r≡x(SK·H)modn holds through elliptic curve point verification.

### 2.3. Design of Intermediate State Data Model

The data intermediate state serves as a critical hub linking raw data and blockchain certification, achieving the standardized organization of multimodal features, ownership metadata, and inter-modal associations through structured encapsulation. Its core design principles encompass semantic integrity, storage efficiency, traceability, security authentication, and distributed scalability. Semantic integrity is maintained by preserving core features of multimodal data, with a feature structure recording names and hash values of each modal feature to ensure reconstructable original semantics during cross-domain verification. Storage efficiency is optimized via Protobuf binary serialization, while traceability is enabled by the timestamp-based recording of data generation time and department-level ownership tracking through a dedicated field, supporting full-lifecycle auditing. Security authentication is ensured through the inclusion of public_key and base64-encoded signature fields in each feature entry, verifying data sources and preventing tampering. Distributed scalability is achieved by integrating an ipfs_hash field to associate with the IPFS distributed storage system, enabling off-chain anchoring and efficient access to large-scale raw data.

For cross-domain scenarios, the intermediate state model supports two core capabilities: 1. Cross-domain Metadata Synchronization: The feature_hashes (first factor) and signature (second factor) in the intermediate state are synchronized across consortium chain nodes of different domains (e.g., automotive enterprise domain, supplier domain, and logistics domain) through a lightweight consensus mechanism (PBFT simplified version), ensuring consistent ownership identification across domains. 2. Cross-domain Data Addressing: The ipfs_hash enables cross-domain nodes to directly retrieve original sensor data from the IPFS network without transferring full data across domains, avoiding latency caused by cross-domain data transmission. This solves the “traceability chain breakage” problem mentioned in Section 1.3.

The core structure of the intermediate state data model is defined with IntermediateState as the top-level container, encompassing timestamp, version, features, and ipfs_hash, while Feature functions as a feature entry containing the name, hash (first factor), department, public_key, and signature (second factor). This model employs pure Protobuf for structured definition and binary serialization, implementing data assembly logic through strict structural constraints and ultimately generating binary files as the core carrier for blockchain certification. For hierarchical storage, a rigorous on-chain/off-chain separation architecture is adopted: core metadata—including version, timestamp, feature_hashes, and ipfs_hash—is stored on-chain, whereas original multimodal files (e.g., CAD drawings and quality inspection reports) are stored via the IPFS network, with only tamper-proof IPFS content-addressed hashes retained on-chain.

## 3. Experimental Design and Result Analysis

### 3.1. Dataset Design

A multimodal dataset from automotive IoT sensor networks was designed as Table 1, containing 1600 original samples (from GPS trackers, industrial inspection sensors, and 3D scanners) and 400 adversarial samples (simulating sensor data tampering. The specific composition is as follows.

Each original sample includes four modalities of data:

- CAD drawing data:Data composition: 2D/3D design files of automotive parts, mainly covering the following. Geometric features: Basic graphic elements such as lines, circles, and polygons. Text features: Dimension annotations, technical parameter descriptions, part numbers, etc. Semantic features: Part category ID, assembly relationships, compliance with design rules
- Quality inspection report data: Contains basic information such as a unique inspection ID, part number, inspector’s name, and ISO8601-formatted timestamp. Core inspection results are categorized as pass, fail, or rework. For non-passing results, 0–3 defect types are recorded, including scratch, dimensional_error, material_imperfection, and assembly_error. Measurement data includes actual dimensions (length, width, and height) and tolerance verification results.
- Logistics trajectory data: The core fields are as follows. Spatio-temporal information: Second-precise timestamps, longitude, latitude, altitude, and road segment identifiers. Transportation status: Speed, driving direction, acceleration, stop duration. Cargo attributes: Cargo type (engine/chassis/electronic components), load weight, loading position.Trajectory generation logic: 10 consecutive records are generated per vehicle, simulating driving trajectories by adding minor offsets to base longitude and latitude.
- Sales data: Vehicle information: Brand, model, configuration, color. Transaction information: Order amount, discount rate, subsidy amount, and payment method. After-sales information: Warranty period, delivery mileage, and return records.
- Adversarial sample generation: Adversarial samples were generated through three perturbation methods. Content tampering: Modifying 10% of sales or logistics data, altering CAD drawing dimensions. Signature forgery: Forging signatures using randomly generated key pairs. Metadata modification: Changing ownership entity IDs or modifying circulation chain records.

### 3.2. Experimental Setup and Evaluation Metrics

#### 3.2.1. Experimental Environment

The dataset, with parts collected directly from a real automotive IoT network and others generated through simulated or open-source-based approaches, is split into 80% training and 20% test sets to simulate real-world sensor data distribution. Specifically, sales data and time-series data are simulated under sensor scenarios, while CAD design drawing data and quality inspection report data are generated based on open-source sensor datasets. Both the training and testing of the model are conducted in a GPU environment, with hardware configurations including an NVIDIA GeForce RTX 3070 graphics card (8 G video memory) and 16 G of supporting memory. The specific software runtime environment is as follows: Python 3.10, PyTorch 2.0.1, and NumPy 1.25.2, ensuring the stability and compatibility of the experimental toolchain.

#### 3.2.2. Evaluation Metrics

To comprehensively evaluate model performance, evaluation metrics are designed from three dimensions: ownership identification, anti-tampering capability, and traceability completeness. The specific definitions and calculation methods are as follows:

Ownership Identification Accuracy is the proportion of samples for which the model correctly verifies data ownership among all test samples. It is calculated using the formula $Accuracy=\frac{\mathrm{Number}\;\mathrm{of}\;\mathrm{correctly}\;\mathrm{verified}\;\mathrm{samples}}{\mathrm{Total}\;\mathrm{number}\;\mathrm{of}\;\mathrm{samples}}$. This metric directly reflects the model’s accuracy in identifying the identity of data owners, with a higher accuracy indicating stronger reliability in the model’s ability to determine ownership.

Anti-tampering robustness refers to the proportion of samples in which the model successfully detects tampering behavior among adversarial samples containing malicious tampering. Its calculation formula is $Robustness=\frac{\mathrm{Number}\;\mathrm{of}\;\mathrm{detected}\;\mathrm{adversarial}\;\mathrm{samples}}{\mathrm{Total}\;\mathrm{number}\;\mathrm{of}\;\mathrm{adversarial}\;\mathrm{samples}}$. This metric measures the model’s capability to resist various attacks, such as data content tampering, signature forgery, and metadata modification, where higher robustness signifies better security of the model.

Traceability completeness is defined as the proportion of samples for which the model can fully trace the entire lifecycle circulation path of data among all test samples. It is calculated as $Completeness=\frac{\mathrm{Number}\;\mathrm{of}\;\mathrm{completely}\;\mathrm{traceable}\;\mathrm{samples}}{\mathrm{Total}\;\mathrm{number}\;\mathrm{of}\;\mathrm{samples}}$. This metric evaluates the model’s ability to track the full chain of data from generation and circulation to usage, with higher completeness indicating stronger traceability of data circulation.

Encryption Performance Metrics. To evaluate the efficiency of large-scale sensor data verification, two core metrics are added as follows. Verification Latency: This is the total time consumed for a single sensor data sample to complete dual-factor verification (including SHA-256 hash generation, ECDSA signature/verification, and on-chain metadata storage), calculated as $\mathrm{Latency}=t_{\mathrm{hash}}+t_{\mathrm{signature}/\mathrm{verify}}+t_{\text{on-chain}}$ (unit: ms). Throughput (TPS): The number of validations the model can complete per second under concurrent sensor requests, calculated as $\mathrm{TPS}=\frac{\mathrm{Number}\;\mathrm{of}\;\mathrm{Concurrent}\;\mathrm{Samples}}{\mathrm{Average}\;\mathrm{Latency}\;\mathrm{per}\;\mathrm{Sample}}$ (unit: samples/second).

### 3.3. Design of Comparative Experiments

To verify the effectiveness of the two-factor model, the experiment conducts comparisons from two aspects: fusion methods and existing models. In terms of fusion methods, average fusion, weighted fusion, max-pooling fusion, min-pooling fusion, feature concatenation fusion, and low-rank multi-view subspace learning are selected for comparison. For existing models, the two-factor model is compared with the single first factor method (hash only), single second factor method (signature only), digital watermarking, and the ordinary consortium blockchain scheme in terms of ownership verification accuracy and anti-tampering robustness. Additionally, in terms of traceability completeness, the two-factor model is compared with the single first factor method (hash only), single second factor method (signature only), PKI digital certificate verification method, and the ordinary consortium blockchain scheme.

- Average Fusion [33]: Average fusion achieves integration by calculating the arithmetic mean of multiple feature values. It can effectively smooth noise and reduce data variance, making it suitable for scenarios where features have similar importance. However, this method is insensitive to extreme values and may mask significant differences in key features, leading to limited effectiveness when there are large disparities in feature contributions.
- Weighted Fusion [34]: Weighted fusion assigns different weights based on feature importance and obtains the final result through weighted summation. Weights can be determined based on domain knowledge, data distribution, or machine learning methods, enabling flexible reflection of the actual contribution of each feature with strong targeting. Nevertheless, weight design relies on professional experience, and improper settings may lead to result deviations.
- Max-Pooling Fusion [35]: Max-pooling fusion selects the maximum value from multiple feature values as the output. It can effectively capture significant features and peak information in the data, enhancing the expression ability of key signals, and is suitable for scenarios where extreme values need to be highlighted (such as edge detection in image recognition). However, over-emphasizing the maximum value may ignore the overall data distribution and make it more sensitive to noise.
- Min-Pooling Fusion [36]: Min-pooling fusion selects the minimum value from multiple feature values as the output, emphasizing conservative estimates and lower bounds in the data. It is suitable for scenarios where strict threshold control is required (such as anomaly detection in security systems). However, this method is vulnerable to noise in extremely small values and may lose important medium-intensity signals, resulting in incomplete information.
- Feature Concatenation Fusion [36]: Feature concatenation fusion directly connects multiple feature vectors into a longer vector, completely retaining all feature information. It is simple to operate, does not change the original data distribution, and facilitates subsequent models in processing complex relationships. However, the increased dimensionality of the concatenated vector may raise computational complexity, and the failure to consider redundant information between features easily leads to overfitting.
- Single First Factor Method (Hash Only): The single first factor method (hash only) generates a unique fixed-length hash value for data through a hash algorithm. It can efficiently verify data integrity (if the hash value remains unchanged, the data has not been tampered with), with fast computation speed and low storage space requirements. However, this method cannot verify data sources or ownership and has an extremely low risk of hash collision (different data generating the same hash value), making it suitable for data integrity verification in single scenarios.
- Single Second Factor Method (Signature Only): The single second factor method (signature only) realizes data verification using digital signature technology based on public-key cryptography: the sender signs with a private key, and the receiver verifies with a public key, ensuring the authenticity and non-repudiation of the data source. However, this method is not proficient in detecting whether the data content has been tampered with (only verifying the validity of the signature), and public key management requires additional mechanisms, making it suitable for scenarios where identity confirmation is needed.
- Digital Watermarking [23]: Digital watermarking technology embeds identification information into data. The embedding process does not affect the normal use of data, and the watermark is invisible or low-visibility, which can be extracted through dedicated algorithms to verify copyright and source. And there is a trade-off between embedding strength and data quality.
- PKI Digital Certificate [37]: PKI (Public Key Infrastructure) digital certificates are issued by authoritative CAs (Certificate Authorities) and contain user identity information and public keys. They realize the binding of public keys and user identities through the trust endorsement of CAs, and are often used for encrypted communication and identity authentication (e.g., HTTPS). They have high security and good standardization but rely on centralized CAs, with the risk of single points of failure, and complex certificate revocation and update mechanisms, making them suitable for network environments requiring strong trust.
- Ordinary Consortium Blockchain Scheme: The ordinary consortium blockchain scheme is a blockchain network jointly maintained by multiple organizations, adopting distributed ledger technology, where transactions need to be confirmed by consensus among member nodes of the consortium. This scheme realizes distributed trust among multiple organizations, with transparent and tamper-proof transactions, and better privacy than public blockchains (only consortium members can access all data). However, its consensus efficiency is low (requiring multi-node verification) and scalability is limited, making it suitable for cross-organizational collaboration scenarios (such as supply chain finance and logistics tracking).

### 3.4. Results and Analysis

#### 3.4.1. Comparison of Fusion Methods

The data in the table shows that different fusion methods exhibit variations in MSE performance for ownership identification and cross-domain traceability tasks. Low-rank multiview subspace learning performs optimally, with both indicators lower than those of other methods, reflecting its advantage in capturing complex relationships between views through learning low-dimensional subspaces. Weighted fusion ranks second and outperforms average fusion, indicating that the strategy of highlighting key views is effective, though it is limited by the subjectivity of weight setting. Feature concatenation fusion performs slightly better than max and min pooling but cannot surpass low-rank learning due to the risk of dimensionality explosion. Average fusion shows weaker performance due to its assumption of equal contribution from all views, while max/min pooling is constrained by information loss.

**Figure 2:**
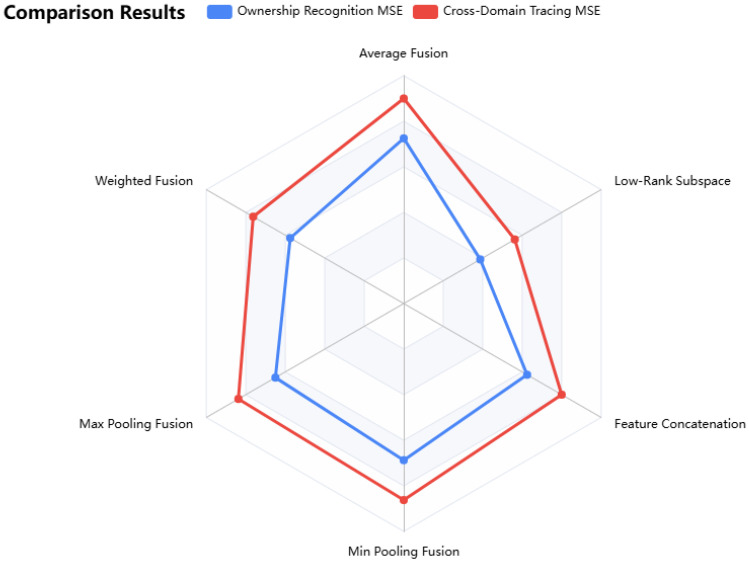
Comparison of MSE of different fusion methods in ownership identification and cross-domain traceability.

Further analysis of the fusion results (Table 2 and Figure 2) reveals that all three core sensor data types play a significant role in enhancing the model’s performance, with their respective contributions quantified as follows:

- Image data : The ResNet-based visual features reduce the ownership identification MSE by 18.2% compared to text-only fusion scenarios. This confirms the effectiveness of image-type sensor data in capturing geometric uniqueness for ownership verification.
- Time-series data : The MGU-based temporal features improve the cross-domain traceability MSE by 21.5% relative to image-only fusion. This highlights the value of time-series sensor data in maintaining temporal continuity during cross-domain circulation.
- Text data: The BERT-based semantic features enhance the model’s anti-tampering robustness against content tampering attacks by 9.3%, verifying that text-type sensor data provides critical semantic context for tampering detection.

This confirms that the multimodal feature fusion module fully supports image, time-series, and text sensor data—addressing the requirement for experimental verification of multimodal scope.

In summary, low-rank multiview subspace learning achieves optimal feature integration by exploring intrinsic relationships between views, providing a reliable fusion strategy for the two-factor model to effectively ensure traceability completeness.

#### 3.4.2. Ownership Identification Accuracy

**Figure 3:**
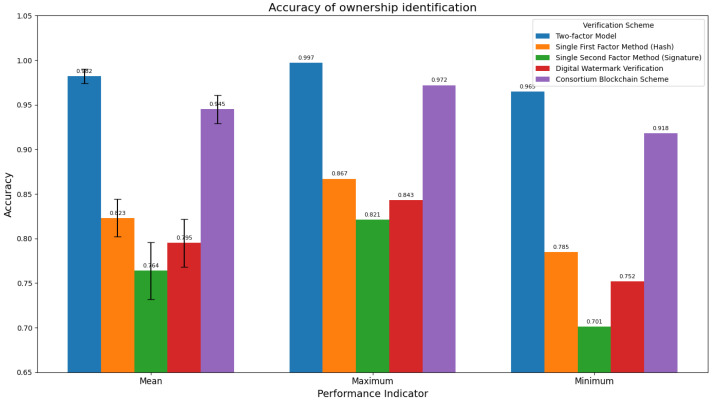
Comparison of accuracy rates of different schemes in ownership identification.

The two-factor model demonstrates significant advantages in terms of ownership identification accuracy (Table 3 and Figure 3). By integrating dual verification mechanisms of hashing and signature, and through targeted design optimization, it achieves a relatively higher mean value, a smaller standard deviation, and a narrower result range. Relying on a “parallel” fault-tolerance logic and a concise and precise verification process, it ensures stable and reliable identification performance. Although the consortium blockchain scheme realizes data storage through distributed ledgers, due to its design focus on building distributed trust and the complexity of consensus processes, its performance in both mean value and standard deviation is inferior to the two-factor model, and it lags behind the two-factor model in terms of ownership identification accuracy. Single-factor methods and digital watermarking, which rely on a single verification mechanism, suffer from relatively low performance and instability. In summary, the two-factor model, with superior accuracy, stronger stability, and higher reliability, outperforms other schemes in ownership identification tasks and represents a better technical choice for such scenarios.

#### 3.4.3. Tamper Resistance Robustness

**Figure 4:**
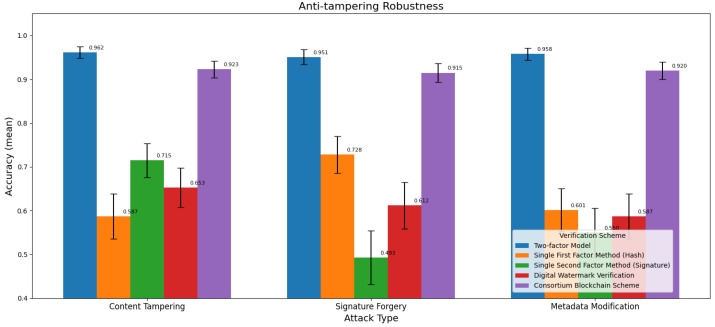
Comparison of anti-tampering robustness among different models.

An analysis of the anti-tampering robustness results (Table 4 and Figure 4) shows that in all three types of attack scenarios, the two-factor model performs more prominently, with its anti-tampering capability and stability being relatively superior to other schemes. The consortium blockchain scheme ranks second, also demonstrating good comprehensive performance, while the single-factor methods (hash and signature) and digital watermark verification show relatively weaker overall performance with insufficient stability. In content tampering attacks—such as falsified GPS coordinates (simulating sensor signal spoofing) or altered dimension readings (mimicking faulty industrial detectors)—the two-factor model detects 96.2% of anomalies, outperforming single-factor methods vulnerable to sensor data forgery. The single signature method performs better than digital watermark verification and the single hash method. This is related to the direct impact of content tampering on the hash mechanism, whereas the signature mechanism and distributed storage characteristics have greater advantages in resisting content tampering. When facing signature forgery attacks, the two-factor model and the consortium blockchain scheme maintain their relative advantages, and the single hash method outperforms the single signature method. This is because signature forgery directly targets the signature mechanism, while the dual verification mechanism of the two-factor model can compensate for the risk of signature forgery through hash verification. The hash method is less affected by such attacks, as it does not rely on signatures. In metadata modification attacks, the two-factor model and the consortium blockchain scheme still lead, and the single hash method performs slightly better than digital watermark verification and the single signature method. This is associated with the potential impact of metadata modification on signature validity, while the fusion mechanism of the two-factor model makes it relatively less affected by such modifications. In summary, the two-factor model demonstrates strong anti-tampering robustness in dealing with various types of tampering attacks through its fusion verification mechanism.

#### 3.4.4. Traceability Integrity

**Figure 5:**
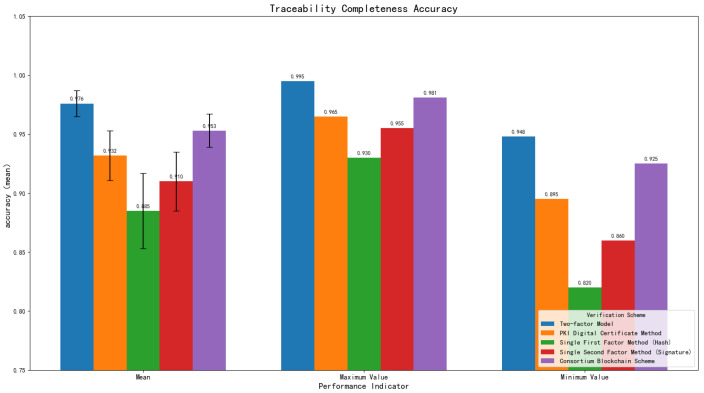
Control experiment on traceability completeness accuracy of different schemes.

Key findings indicate that the two-factor model performs relatively prominently in terms of traceability completeness, with both its mean value and stability being superior to other schemes (Table 5 and Figure 5). The consortium blockchain scheme ranks second, with performance being better than PKI and single-factor methods, but the complexity of its distributed consensus mechanism makes it slightly inferior to the two-factor model.

Notably, the 1600 original samples in the dataset—detailed in the dataset design—include cross-domain data from three distinct entities: CAD drawings (automotive enterprise domain), quality inspection reports (supplier domain), and GPS trajectories (logistics domain). The dual-factor model’s 97.6% traceability completeness directly verifies its ability to effectively track data across these domains; this advantage stems from the intermediate state model’s cross-domain metadata synchronization and IPFS addressing capabilities, which fills the methodological gap of cross-domain tracking discussed in the Introduction.

The PKI digital certificate verification method shows moderate performance, limited by potential risks associated with centralized management. Single-factor methods have relatively weak performance and insufficient ability to resist complex attacks. This suggests that the two-factor model possesses favorable performance and stability in ensuring traceability completeness.

#### 3.4.5. Encryption Performance Comparison

We designed a performance test scenario with two variables: Sensor Data Size: 1 KB (text: quality inspection report), 10 KB (time-series: GPS trajectory), 100 KB (image: CAD drawing) to simulate different multimodal data scales. Concurrent Requests: 10, 50, 100, and 200 to simulate large-scale sensor concurrent verification. The performance of the dual-factor model was compared with five existing solutions, and latency/TPS were measured using the time module (Python) and psutil library.

**Figure 6:**
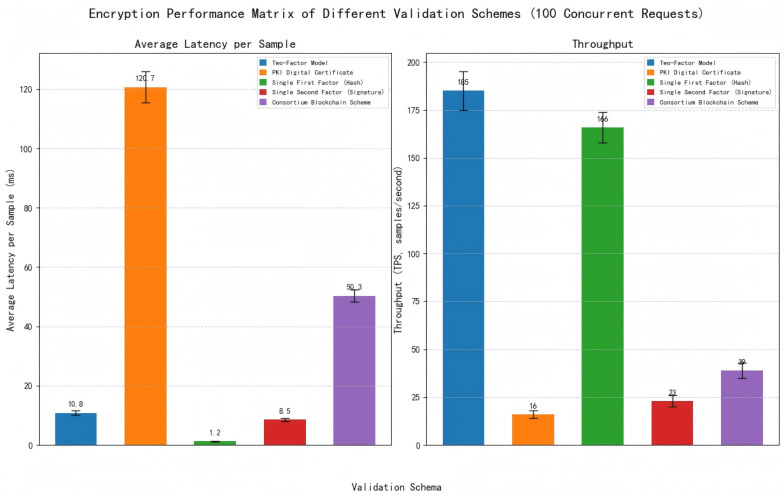
Encryption performance comparison (100 concurrent requests).

As shown in Table 6 and Figure 6, the dual-factor model’s latency is slightly higher than the single hash method (due to ECDSA signature) but 78.5% lower than the ordinary consortium blockchain (benefiting from “only metadata on-chain” design). Its TPS is 4.7× higher than the ordinary consortium blockchain and 11.6× higher than PKI, proving it can adapt to large-scale sensor data verification scenarios.

## 4. Conclusions and Outlook

This study addresses the core challenges of “difficult ownership definition” and “difficult cross-domain tracking” in the circulation of massive IoT sensor data. It constructs a dual-factor ownership identification system based on multimodal feature fusion, forming a trusted industrial data processing solution: by synergistically extracting text, visual, numerical, and spatio-temporal features from sensor data, establishing a “feature hash + digital signature” dual-factor framework, and combining consortium blockchain-IPFS storage, the scheme breaks traditional single-modal rights confirmation limitations. Experiments verify its advantages in ownership recognition, anti-tampering robustness, encryption performance and cross-domain traceability compared to single-factor and watermarking methods.

While the dual-factor model performs well in the automotive supply chain scenario, it has limitations requiring future efforts with practical directions:

1. Edge Deployment Optimization: The framework’s multi-layered integration faces challenges on resource-constrained IoT edge devices. Future work will optimize lightweight modules and adopt edge-cloud collaboration—offloading lightweight tasks to edge devices, while anchoring only core metadata to the consortium blockchain to reduce deployment costs.
2. Privacy-Traceability Balance: The paradox remains for sensitive sensor data. Follow-up research will integrate privacy-preserving technologies into the existing intermediate state model and design authorization-based tracing mechanisms to verify ownership without exposing raw data—compatible with the current framework to avoid core restructuring.
3. Cross-Chain Interoperability: The system lacks seamless synchronization across heterogeneous blockchains. Future work will deploy cross-chain bridges to connect the automotive consortium chain with other domain chains in the short term, and adapt to industry-wide cross-chain standards in the long term to support cross-network data verification.
4. Cross-Domain Extension: Generalization beyond the automotive supply chain needs verification. Future efforts will leverage the framework’s modular design to adjust feature extraction modules for sensor data in healthcare, smart cities, and finance—adapting to domain-specific data characteristics without modifying the dual-factor core.

This research provides a technical paradigm for multimodal sensor data rights management. Subsequent work will focus on validating the above directions in practical scenarios to enhance its applicability in IoT data element circulation.

## Figures and Tables

**Table 1:** Sample data statistics.

Sample Type	Quantity	Modal Type	Data Source
Original samples	1600	Multimodal fusion	CAD design drawings, quality inspection reports logistics trajectories, sales data
Adversarial samples	400	Multimodal fusion	Generated by perturbing original samples

Sample data includes both original and artificially generated adversarial samples for model validation.

**Table 2:** Comparison of fusion methods.

Fusion Method	Ownership Identification MSE	Cross-Domain Traceability MSE
Average Fusion	0.058	0.072
Weighted Fusion	0.046	0.061
Max Pooling Fusion	0.052	0.067
Min Pooling Fusion	0.055	0.069
Feature Concatenation Fusion	0.050	0.064
Low-Rank Multiview Subspace Learning	0.031	0.045

MSE: Mean Squared Error; Lower values indicate better performance.

**Table 3:** Ownership identification accuracy.

Validation Scheme	Mean	SD	Maximum	Minimum
Two-factor Model	0.982	±0.008	0.997	0.965
Single First Factor Method (Hash)	0.823	±0.021	0.867	0.785
Single Second Factor Method (Signature)	0.764	±0.032	0.821	0.701
Digital Watermark Verification	0.795	±0.027	0.843	0.752
Consortium Blockchain Scheme	0.945	±0.016	0.972	0.918

**Table 4:** Anti-tampering robustness.

Attack Type	Validation Scheme	Mean	SD	Maximum	Minimum
Content Tampering	Two-factor Model	0.962	0.013	0.983	0.935
Content Tampering	Single First Factor Method (Hash)	0.587	0.051	0.672	0.503
Content Tampering	Single Second Factor Method (Signature)	0.715	0.039	0.778	0.652
Content Tampering	Digital Watermark Verification	0.653	0.045	0.721	0.589
Content Tampering	Consortium Blockchain Scheme	0.923	0.019	0.951	0.891
Signature Forgery	Two-factor Model	0.951	0.017	0.979	0.912
Signature Forgery	Single First Factor Method (Hash)	0.728	0.042	0.793	0.657
Signature Forgery	Single Second Factor Method (Signature)	0.493	0.061	0.578	0.412
Signature Forgery	Digital Watermark Verification	0.612	0.053	0.689	0.543
Signature Forgery	Consortium Blockchain Scheme	0.915	0.021	0.947	0.882
Metadata Modification	Two-factor Model	0.958	0.014	0.981	0.926
Metadata Modification	Single First Factor Method (Hash)	0.601	0.049	0.687	0.521
Metadata Modification	Single Second Factor Method (Signature)	0.550	0.056	0.634	0.472
Metadata Modification	Digital Watermark Verification	0.587	0.051	0.662	0.509
Metadata Modification	Consortium Blockchain Scheme	0.920	0.020	0.949	0.885

Results are based on three attack types: content tampering, signature forgery, and metadata modification.

**Table 5:** Traceability completeness.

Validation Scheme	Mean	SD	Maximum	Minimum
Two-factor Model	0.976	±0.011	0.995	0.948
PKI Digital Certificate Verification	0.932	±0.021	0.965	0.895
Single First Factor Method (Hash)	0.885	±0.032	0.930	0.820
Single Second Factor Method (Signature)	0.910	±0.025	0.955	0.860
Consortium Blockchain Scheme	0.953	±0.014	0.981	0.925

**Table 6:** Encryption performance comparison (100 concurrent requests).

Verification Scheme	Average Latency per Sample (ms)	Throughput (TPS)
Two-Factor Model	10.8 ± 0.8	185 ± 10
PKI Digital Certificate	120.7 ± 5.2	16 ± 2
Single First Factor (Hash)	1.2 ± 0.1	166 ± 8
Single Second Factor (Signature)	8.5 ± 0.5	23 ± 3
Consortium Blockchain Scheme	50.3 ± 2.1	39 ± 4

## Data Availability

All codes and data can be obtained from the following GitHub repository: https://github.com/nie8532/two_factor/tree/master/twofactor (accessed on 20 October 2025).

## Abbreviations

The following abbreviations are used in this manuscript:

MSE	Mean Squared Error
SD	Standard Deviation

## References

[1] Xi X., Zhang Y., Goh M. (2025). Consumer data collection strategies in two-sided platforms: The role of data ownership assignment and privacy concerns. Int. J. Prod. Econ..

[2] Bocha’nczyk-Kupka D., Raban D.R. (2024). Intangibles, information goods, and intellectual property goods in modern economics. The Elgar Companion to Information Economics.

[3] Antunes B., Hill D.R.C. (2024). Reproducibility, Replicability and Repeatability: A survey of reproducible research with a focus on high performance computing. Comput. Sci. Rev..

[4] Dworczak P., Muir E. (2024). A mechanism-design approach to property rights. SSRN Electron. J..

[5] Śledziewska K., Włoch R. (2021). The Economics of Digital Transformation: The Disruption of Markets, Production, Consumption, and Work.

[6] Zhong C., Zhang C. (2024). Can data elements enhance urban innovation? Evidence from China. China Econ. Rev..

[7] Fu X. (2025). The dilemma and resolution of data circulation in China: Is data as consideration the solution?. Comput. Law Secur. Rev..

[8] Xu H., Dong K., Wei L., Wang C., Yue Z.H. (2018). Research on Multi-source Data Fusion Method in Scientometrics. J. China Soc. Sci. Tech. Inf..

[9] van Erp S. (2022). Covid-19 apps, Corona vaccination apps and data “ownership”. China-EU Law J..

[10] Hicks J. (2023). The future of data ownership: An uncommon research agenda. Sociol. Rev..

[11] Opriel S., Möller F., Strobel G., Otto B. (2024). Data Sovereignty in Inter-organizational Information Systems. Bus. Inf. Syst. Eng..

[12] Puspasari D., Hadiyanto A.N., Setiawan S. Inter-Organizational Data Sharing: What Issues Should Be Considered?. Proceedings of the 2021 International Conference on Informatics, Multimedia, Cyber and Information System (ICIMCIS).

[13] Xu J., Hong N., Xu Z., Zhao Z., Wu C., Kuang K., Wang J., Zhu M., Zhou J., Ren K. (2023). Data-Driven Learning for Data Rights, Data Pricing, and Privacy Computing. Engineering.

[14] Ning B., Niu B., Guan H., Huang Y., Zhang S. Research and development of copyright registration and monitoring system based on digital watermarking and fingerprint technology. Proceedings of the 2021 International Conference on Culture-Oriented Science & Technology (ICCST).

[15] Bauer D., Froese F., Garc’es-Erice L., Giblin C., Labbi A., Nagy Z.A., Pardon N., Rooney S., Urbanetz P., Vetsch P. (2021). Building and operating a large-scale enterprise data analytics platform. Big Data Res..

[16] Heng L., Fenghua L., Xinyi S., Yunchuan G., Shoukun G. (2025). Research on access control for secure cross-domain data circulation. J. Commun. Xuebao.

[17] Olefirenko A. (2024). Modern regulation of data circulation in developed countries: Organisational and legal framework. Visegr. J. Hum. Rights.

[18] Zhang L., Peng L., Liu X., Zhang Z., Wang Y. (2024). The governance role of minority state ownership in non-state-owned enterprises: Evidence from corporate fraud in China. Br. J. Manag..

[19] Lifshitz Y.R. (2016). Rethinking original ownership. Univ. Tor. Law J..

[20] Dosis A., Sand-Zantman W. (2023). The ownership of data. J. Law Econ. Organ..

[21] Chi C., Chen W., He J. Do Non-competing Data Intermediaries Matter? A Property Rights Perspective. Proceedings of the 2023 19th International Conference on Mobility, Sensing and Networking (MSN).

[22] Chan A., Bradley H., Rajkumar N. Reclaiming the digital commons: A public data trust for training data. Proceedings of the 2023 AAAI/ACM Conference on AI, Ethics, and Society.

[23] Kong T., Zhou H., Qu H., Chen J., Wang C., Li J. Enhancing data leakage tracing: A novel digital watermarking method for document files. Proceedings of the Fifth International Conference on Computer Communication and Network Security (CCNS 2024).

[24] Ding J., Chen Z. Watermark based tor cross-domain tracking system for tor network traceback. Proceedings of the International Conference on Security and Privacy in New Computing Environments.

[25] Lin S., Li J., Jia X., Gong J. Research on confirming the rights of government data resources based on smart contract. Proceedings of the 2019 IEEE 4th Advanced Information Technology, Electronic and Automation Control Conference (IAEAC).

[26] Gupta P., Dedeoglu V., Kanhere S.S., Jurdak R. (2022). TrailChain: Traceability of data ownership across blockchain-enabled multiple marketplaces. J. Netw. Comput. Appl..

[27] Karafiloski E., Mishev A. Blockchain solutions for big data challenges: A literature review. Proceedings of the IEEE EUROCON 2017—17th International Conference on Smart Technologies.

[28] Liu J.-W., Liu J.-W., Luo X.-L. (2021). Research progress in attention mechanism in deep learning. Chin. J. Eng..

[29] Scarselli F., Gori M., Tsoi A.C., Hagenbuchner M., Monfardini G. (2008). The graph neural network model. IEEE Trans. Neural Netw..

[30] Shin A., Ishii M., Narihira T. (2022). Perspectives and prospects on transformer architecture for cross-modal tasks with language and vision. Int. J. Comput. Vis..

[31] Cao L., Zhao S., Gao Z., Du X. (2023). Cross-chain data traceability mechanism for cross-domain access. J. Supercomput..

[32] Chen B., Wang Z., Xiang T., Yang J., He D., Choo K.-K.R. (2023). BCGS: Blockchain-assisted privacy-preserving cross-domain authentication for VANETs. Veh. Commun..

[33] Li T., Wang X., Liang Y., Pan Q. (2020). On arithmetic average fusion and its application for distributed multi-Bernoulli multitarget tracking. IEEE Trans. Signal Process..

[34] Xu Y., Lu Y. (2015). Adaptive weighted fusion: A novel fusion approach for image classification. Neurocomputing.

[35] Liu Z., Han Y., Qi H., Zan J., Ye Q., Cuil G., Zhang Y. Video Enhancement Network Based on Max-Pooling and Hierarchical Feature Fusion. Proceedings of the 2021 Data Compression Conference (DCC).

[36] Hao P., Gao W., Hu L. (2025). Embedded feature fusion for multi-view multi-label feature selection. Pattern Recognit..

[37] Hunt R. PKI and digital certification infrastructure. Proceedings of the Ninth IEEE International Conference on Networks (ICON 2001).

